# Consensus HIV-1 FSU-A Integrase Gene Variants Electroporated into Mice Induce Polyfunctional Antigen-Specific CD4+ and CD8+ T Cells

**DOI:** 10.1371/journal.pone.0062720

**Published:** 2013-05-08

**Authors:** Olga Krotova, Elizaveta Starodubova, Stefan Petkov, Linda Kostic, Julia Agapkina, David Hallengärd, Alecia Viklund, Oleg Latyshev, Eva Gelius, Tomas Dillenbeck, Vadim Karpov, Marina Gottikh, Igor M. Belyakov, Vladimir Lukashov, Maria G. Isaguliants

**Affiliations:** 1 Department of Microbiology, Tumor and Cell Biology, Karolinska Institutet, Stockholm, Sweden; 2 DI Ivanovsky Institute of Virology, Moscow, Russia; 3 WA Engelhardt Institute of Molecular Biology, Moscow, Russia; 4 Mabtech AB, Stockholm, Sweden; 5 Belozersky Institute of Physico-Chemical Biology, Lomonosov Moscow State University, Moscow, Russia; 6 Michigan Nanotechnology Institute for Medicine and Biological Sciences, and the Department of Internal Medicine, University of Michigan, School of Medicine, Ann Arbor, Michigan, United States of America; 7 Department of Medical Microbiology, Academic Medical Center, University of Amsterdam, Amsterdam, The Netherlands; St.Louis University, United States of America

## Abstract

Our objective is to create gene immunogens targeted against drug-resistant HIV-1, focusing on HIV-1 enzymes as critical components in viral replication and drug resistance. Consensus-based gene vaccines are specifically fit for variable pathogens such as HIV-1 and have many advantages over viral genes and their expression-optimized variants. With this in mind, we designed the consensus integrase (IN) of the HIV-1 clade A strain predominant in the territory of the former Soviet Union and its inactivated derivative with and without mutations conferring resistance to elvitegravir. Humanized IN gene was synthesized; and inactivated derivatives (with 64D in the active site mutated to V) with and without elvitegravir-resistance mutations were generated by site-mutagenesis. Activity tests of IN variants expressed in *E coli* showed the consensus IN to be active, while both D64V-variants were devoid of specific activities. IN genes cloned in the DNA-immunization vector pVax1 (pVaxIN plasmids) were highly expressed in human and murine cell lines (>0.7 ng/cell). Injection of BALB/c mice with pVaxIN plasmids followed by electroporation generated potent IFN-γ and IL-2 responses registered in PBMC by day 15 and in splenocytes by day 23 after immunization. Multiparametric FACS demonstrated that CD8+ and CD4+ T cells of gene-immunized mice stimulated with IN-derived peptides secreted IFN-γ, IL-2, and TNF-α. The multi-cytokine responses of CD8+ and CD4+ T-cells correlated with the loss of *in vivo* activity of the luciferase reporter gene co-delivered with pVaxIN plasmids. This indicated the capacity of IN-specific CD4+ and CD8+ T-cells to clear IN/reporter co-expressing cells from the injection sites. Thus, the synthetic HIV-1 clade A integrase genes acted as potent immunogens generating polyfunctional Th1-type CD4+ and CD8+ T cells. Generation of such response is highly desirable for an effective HIV-1 vaccine as it offers a possibility to attack virus-infected cells via both MHC class I and II pathways.

## Introduction

34 million people worldwide are infected with human immunodeficiency virus type 1 (HIV-1) [1]. Highly active antiretroviral therapy (HAART) significantly improves the prognosis for infected individuals but cannot exterminate the virus and in many cases does not suppress the virus load [2]. Furthermore, treatment leads to the development of drug resistance, which initiates the spread of drug-resistant HIV-1 strains. By now, the level of new infections with drug-resistant HIV-1 has reached 15% [3]. Both the acquired drug resistance and primary infections with drug-resistant HIV-1 strains and minority variants grossly limit the therapy options in acute primary as well as chronic HIV-1 infection [4], [5], [6], [7], [8].

Drug-resistant mutations often emerge in highly conserved domains indispensable for protein activity; further mutations in these regions (to mask the new epitopes) are restricted as deleterious to viral viability [9], [10], [11]. Thus, an escape from drugs makes virus vulnerable for the immune system. This is reflected by the changes in the properties of drug-resistant HIV-1 proteins: modified processing and presentation, shifts in the epitope hierarchy, gain of new epitopes, and broadening of HLA-recognition of the mutated regions [12]. This makes drug-resistant HIV-1 proteins quite immunogenic in the natural infection [10], [13], [14]. It is logical to try to use these mutated antigens to induce an immune response against HIV-1 enzymes with the aim to suppress viral replication and limit the development of drug resistance under HAART. Strong immune response induced by drug-resistant HIV-1 antigens in the experimental settings would motivate their incorporation into therapeutic HIV-1 vaccine(s) aimed to support/complement antiretroviral treatment.

Years of HIV-1 vaccine trials and SIV pre-clinical studies showed that the control over viral replication strongly relies on the vaccine’s ability to elicit a multifunctional T cell response against multiple viral targets (multiple HIV and SIV epitopes) [15], [16], [17]. Such response can be effectively generated by genetic vaccination [18]. The latter can induce a protective immune response against viral infections in diverse, also large, species [19], [20], [21], [22], [23]. While early DNA vaccines exploited the genetic material of the microbes, modern vaccines use genetic information to build the synthetic immunogens, often quite different from the microbial genes. Variable pathogens, as HIV-1, are targeted by a specific cluster of synthetic gene vaccines, so called consensus (inferred consensus, ancestral and center-of-tree) immunogens, often more potent than the expression-optimized genes [24], [25], [26], [27]. An encouraging example of their use is the protection against divergent influenza H1N1 viruses after genetic immunization with a Centralized Influenza Hemagglutinin [28]. Several consensus-gene based HIV-1 vaccines have already entered clinical trials [29], [30], [31], [32], [33].

With this in mind, we approached HIV-1 integrase, a key HIV-1 enzyme responsible for provirus integration into the host genome [34]; [35]. Early DNA vaccine trials avoided including HIV-1 integrase genes due to the fear of inducing genomic instability, with the exception of a single trial reporting high immunogenicity of expression-optimized integrase (as a part of the *pol* gene) in rodents and rhesus macaques [36]. Recent HIV-1 multigene vaccine trials included the IN gene but presented no details on the IN gene immunogenicity [37], [38], [39], [40]. This indicated both the feasibility of the IN gene application in preclinical as well as clinical trials, and the need to improve it to achieve better immunogenic performance.

Here, we have designed and tested the prototype immunogens based on the sequence of the wild-type integrase of HIV-1 FSU-A strain and its variant with elvitegravir-conferring mutations, both devoid of the enzymatic activity. All consensus IN gene variants were found to be highly immunogenic in mice.

## Results

### Design of Consensus Integrases

**Figure 1:**
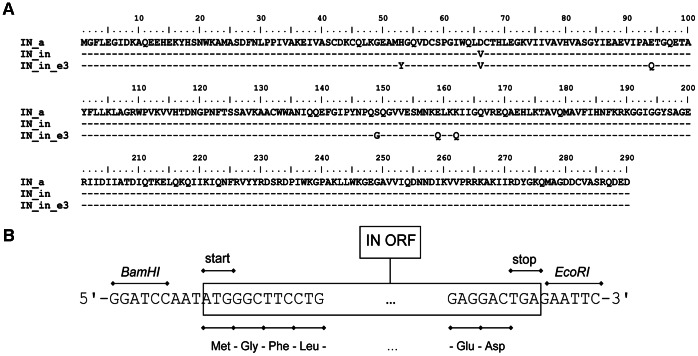
Sequence and structure of the synthetic IN genes. Amino acid sequences of the consensus HIV-1 clade A integrase (IN_a), its inactive variant containing mutation in the active site D64V (IN_in), and inactive variant with mutations conferring resistance to elvitegravir H51Y, E92Q, S147G, E157Q, K160Q (IN_in_e3), all with Met-Gly dipeptide on the N-terminus (**A**); Schematic representation of the structure of the synthetic genes. The following regions are indicated: IN ORF including the Met-Gly dipeptide, 5′- and 3′-end nucleotide flanks with *BamHI* and *EcoRI* restriction sites (**B**).

Full-length sequences of 34 integrase genes of HIV-1 clade A prevalent in the territory of the former Soviet Union including Belarus, Estonia, Georgia, Russia, Ukraine, and Uzbekistan, [[41], [42], [43], [44], and V. Lukashov, unpublished] were translated and aligned, and the amino acid consensus was created. The viral population was very homogeneous with 80% of the consensus fully conserved and an additional 10% having only five ambiguous positions of the total 287 (positions 48, 74, 134, 211, and 218). Consensus integrase sequence was modified to overcome the intrinsic instability due to phenylalanine residue on the N-terminus, which makes IN a physiological substrate of the N-end rule pathway [45], [46]. For this, IN was supplemented with the Met-Gly dipeptide prior to the N-terminal Phe. Extra glycine codon and the triplet ATT upstream of the AUG codon completed the Kozak’s consensus sequence (ANNATGG) required for the efficient initiation of IN gene translation [47]. An inactive form of consensus clade A integrase (IN_in) was created by mutating the first residue of the integrase catalytic triad motif D^64^ to V, as was earlier performed by Cherepanov P. et al [48]. Inactive IN was further supplemented with mutations H51Y, E92Q, S147G, and K160Q, conferring resistance to elvitegravir [49] and a polymorphic mutation E157Q common for subtype A [50], which yielded IN_e3 (D64V+H51Y, E92Q, S147G, E157Q, K160Q). Amino acid sequences of IN variants are presented in Fig. 1.

### Prokarytic Expression and in vitro Activity Tests of the N-terminal His-tagged IN Variants

**Figure 2:**
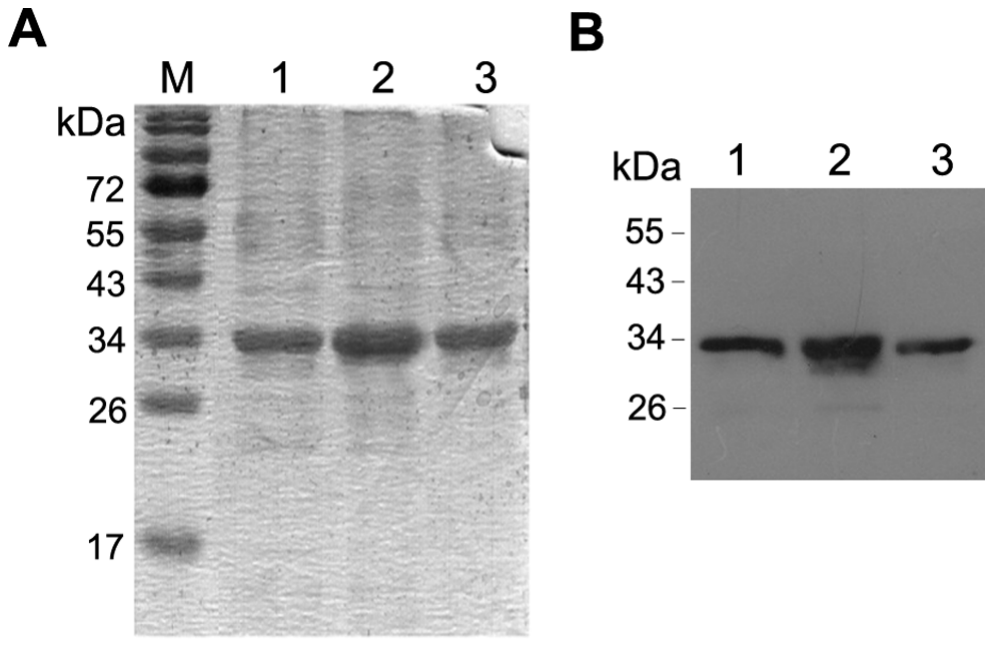
Expression of integrase variants in *E.coli* BL21(DE3). SDS-PAGE analysis of the purified consensus HIV-1 clade A integrase (IN_a, lane 1), its inactivated variant (IN_in, lane 2) and the inactivated IN variant with elvitegravir resistance mutations (IN_in_e3, lane 3) eluted from the Ni-NTA-agarose column with 500 mM imidazole, followed by staining with Coomassie Blue (**A**). Western blotting of integrase preparations (diluted 1∶50) after SDS-PAGE and transfer, using polyclonal rabbit anti-IN antibodies (**B**). Data are representative of three independent experiments.

IN genes cloned into pET15b vector directed high levels of prokaryotic expression of the N-terminal His-tagged IN variants; the levels of prokaryotic IN expression exceeded 10 mg per liter of culture of *E. coli* BL21(DE3) with pRARE plasmid (Fig. S1). His-tagged IN variants were purified by chromatography on the Ni–NTA–agarose to over 80% purity (Fig. 2A). All proteins had the expected molecular mass of 34 kDa and were stained specifically with polyclonal anti-IN antibodies (Fig. 2B).

**Figure 3:**
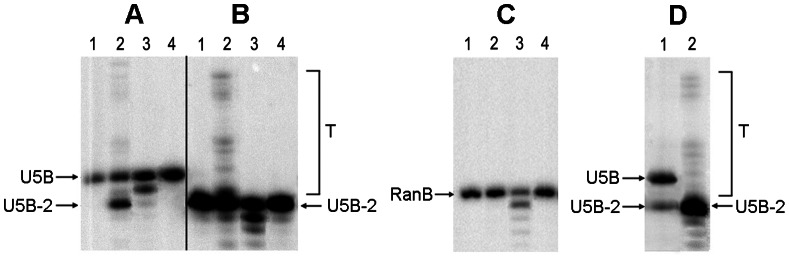
Integrase activities: 3′-processing and DNA strand transfer by IN variants. Products of 3′-processing and strand transfer of the synthetic DNA duplexes with ^32^P-labeled B-strands (Table 1) by the consensus HIV-1 clade A integrase (IN_a), its inactivated variant (IN_in), and the inactivated variant with elvitegravir resistance mutations (IN_in_e3) were separated by gel electrophoresis and quantified using Image-QuantTM 4.1 software. The 3′-processing assay: U5 substrate in the absence of integrases (lane 1) and in the presence of IN_a, IN_in, and IN_in_e3 (lanes 2, 3, and 4, respectively) (**A**). The strand transfer reaction: U5-2 substrate in the absence of integrases (lane 1) and in the presence of IN_a, IN_in, and IN_in_e3 (2, 3, and 4, respectively); T – the strand transfer products (**B**). Incubation of the non-specific DNA Ran in the absence of integrases (lane 1), and in the presence of IN_a, IN_in, and IN_in_e3 (2, 3, and 4, respectively) (**C**). Activities of HXB2 integrase, 3′-processing (1), strand transfer (2) (**D**). Tests were performed with 100 nM integrases and 10 nM DNA. Products were separated in denaturing 20% PAAG with 7M urea (see Methods for details). Data are representative of two independent experiments.

Catalytic activities of the recombinant enzymes were evaluated using standard assays of 3′-processing and strand transfer using ^32^P-labelled oligodeoxyribonucleotide duplexes which mimicked the U5 region of HIV-1 LTR (Table 1). Endonuclease cleavage of the U5 duplex representing 3′-processing resulted in the removal of GT dinucleotide from the 3′-end of the processed strand U5B and formation of the pre-processed oligonucleotide U5B-2. “Self-insertion” of the U5-2 duplex consisting of the pre-processed strand U5B-2 and U5A modeled the reaction of strand transfer (Fig. 3). IN_a performed both reactions with an efficiency higher than that of HBX2 HIV integrase (Fig. 3 A, B compared to D; Table 2). IN_in containing the inactivation mutation D64V could perform neither 3′-processing nor strand transfer, but possessed an exonucleolytic activity (Fig. 3 A, B, C). This activity was sequence-unspecific, since similar digestion patterns were seen after cleavage of the specific substrates U5 and U5-2 (Fig. 3A, B) and of the random DNA duplex (Fig. 3C). IN_in_e3 bearing both inactivation and drug resistance-conferring mutations was inactive (Fig. 3A, B, C; Table 2). To confirm this, IN_in_e3 was incubated with U5 duplex for 24 hours, but neither processing nor non-specific nuclease activities were detected (data not shown).

**Table 1:** Oligonucleotide duplexes used to assess integrase activities.

Abbreviated name	Sequence	Function
U5B	5′-GTGTGGAAAATCTCTAGCAGT-3′*	Strand processed by integrase
U5A	3′-CACACCTTTTAGAGATCGTCA-5′	Complementary to U5B
**U5**	**U5A/U5B duplex**	**Specific integrase substrate in 3′-processing**
U5B-2	5′-GTGTGGAAAATCTCTAGCA-3′	Result of U5B processing
**U5-2**	**U5A/U5B-2 duplex**	**Integrase substrate in the strand transfer reaction**
RanB	5′-GGAATCTAGCGGCGCATAGGT-3′	Complementary to RanA
RanA	3′-CCTTAGATCGCCGCGTATCCA-5′	Complementary to RanB
**Ran**	**RanB/RanA duplex**	**Non-specific DNA duplex**

* The dinucleotide removed by IN is underlined.

**Table 2:** Catalytic activities of the recombinant integrases.

Integrase	3′-processing efficiency, %		Strand transfer efficiency, %	
	measured	relative	measured	relative
**IN (HXB2)**	34±4	100	6,1±0,6	100
**IN_a**	51±5	150±15	7,8±0,8	128±15
**IN_in**	Non-specific nuclease activity	undetectable	undetectable	undetectable
**IN_in_e3**	undetectable	undetectable	undetectable	undetectable

### Expression of Integrases in Eukaryotic Cells

**Figure 4:**
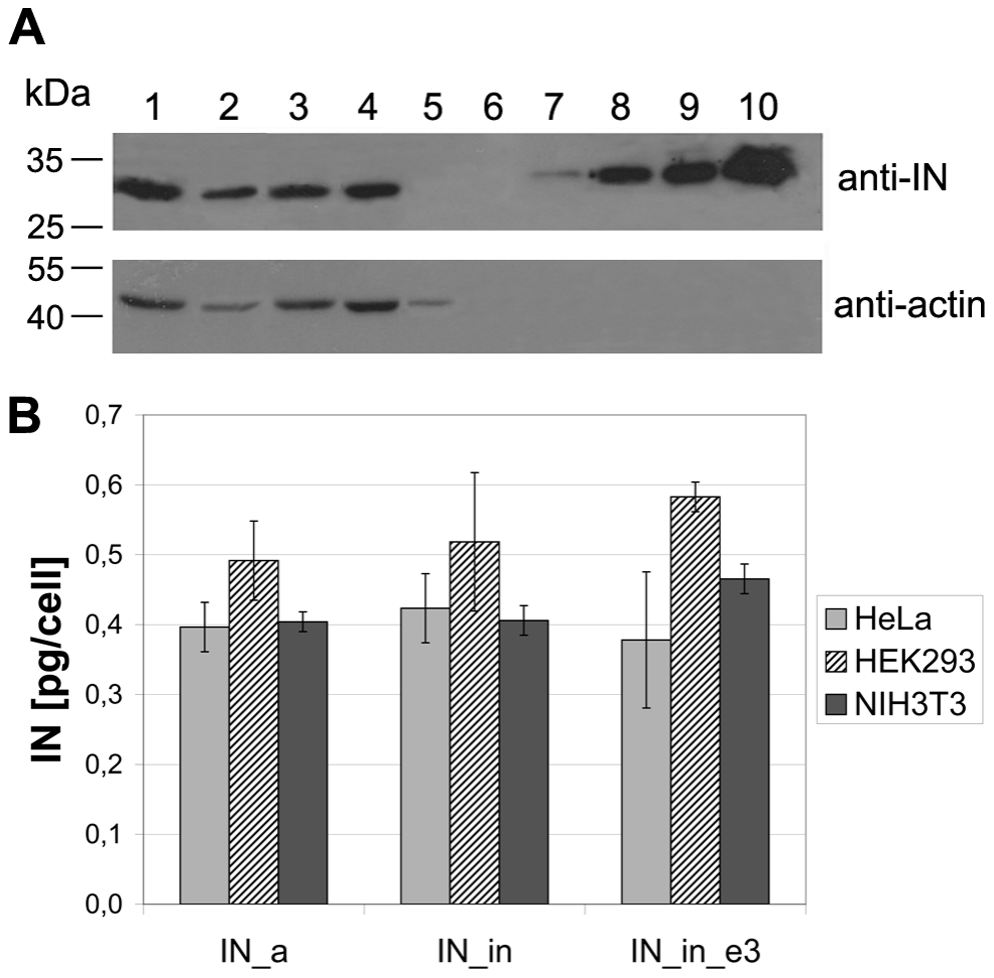
Expression of IN variants in eukaryotic cells. Western blotting of lysates of HeLa cells transfected with the pVaxIN_a (lane 1), pVaxIN_a_e3 (lane 2), pVaxIN_in (lane 3), pVaxIN_in_e3 (lane 4), or empty vector pVax1 (lane 5); recombinant IN of HXB2 carrying 6His-tag (34 kDa) loaded in the amounts of 0.5, 2.5, 5 and 10 ng/well (lanes 7 to 10, respectively). Blot was stained with the rabbit polyclonal anti-IN antibodies, stripped, and re-stained with the monoclonal anti-actin antibodies. Molecular mass markers as defined by the protein ladder (Page Ruler Prestained Protein Ladder, Fermentas; lane 6) are given to the left (**A**). Average amount of the IN variants expressed per transfected human (HeLa, HEK293) or mouse (NIH3T3) cell (results of two independent runs, each done in duplicate) (**B**).

Next, “humanized” IN gene variants were cloned into eukaryotic expression vector pVax1. Human (HeLa, HEK) and mouse (NIH3T3) cell lines transiently transfected with pVaxIN plasmids expressed proteins with the expected molecular mass (33 kDa) specifically stained in Western blots with integrase-specific polyclonal antibodies (Fig. 4A). All IN genes were highly expressed in diverse eukaryotic cell lines (Fig. 4B, and data not shown). Having high expression levels and expected enzymatic properties (active or inactive), they fulfilled the prerequisites for using them as DNA immunogens.

### Integrase Genes in pVax1 Induce Potent Cellular Immune Responses

The immunogenicity of integrase genes was assessed in BALB/c mice. For this, BALB/c mice were subcutaneously injected with pVaxIN variants with subsequent electroporation (for description, see Materials and Methods). Blood was collected on day 15 after immunization, and PBMC were isolated and analyzed by dual IFN-γ/IL-2 Fluorospot for the capacity to secrete IFN-γ, IL-2 and both cytokines in response to stimulation with integrase-derived synthetic peptides. A similar assay was run on mouse splenocytes collected after the completion of immunization on day 22.

**Figure 5:**
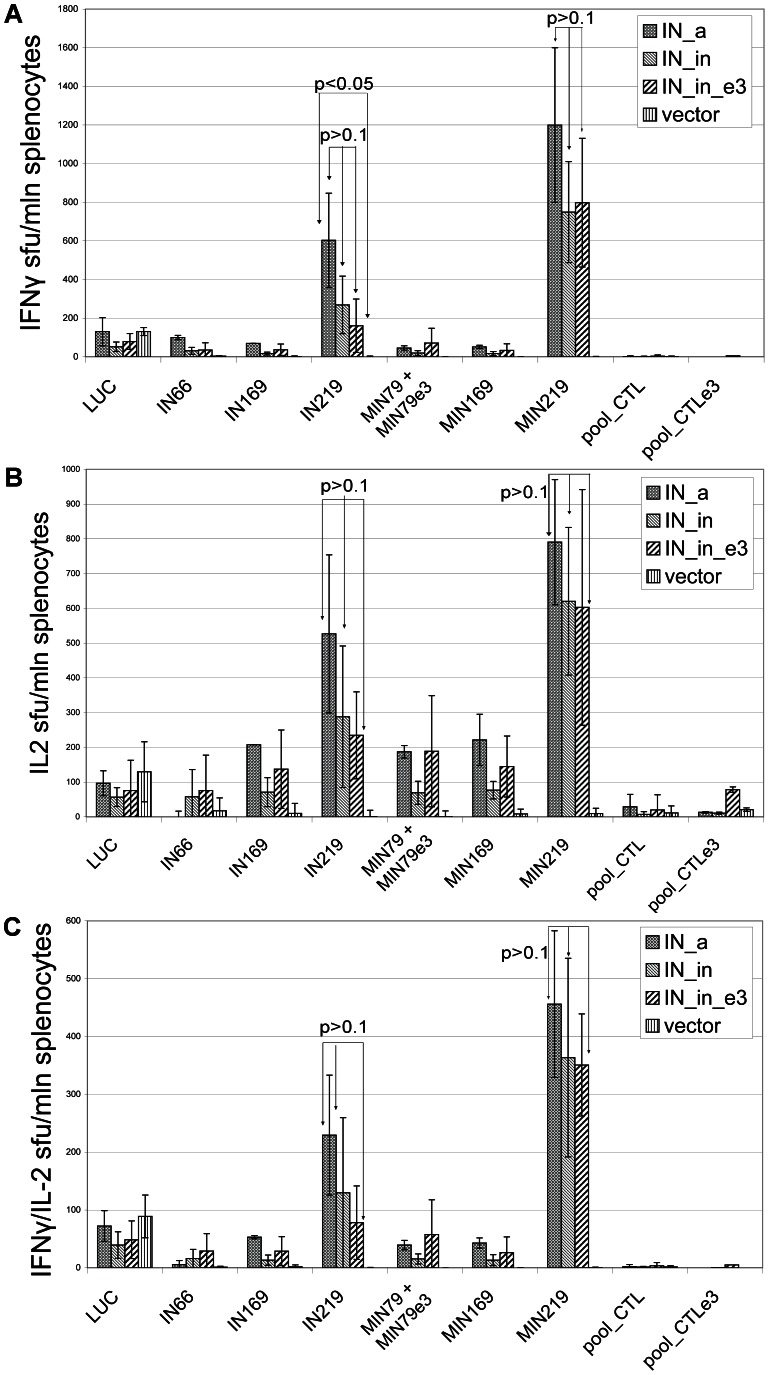
IFN-γ/IL-2 Fluorospot assay of the splenocytes of mice immunized with IN gene variants. The results of IFN-γ/IL-2 Fluorospot performed on splenocytes of mice immunized with plasmids encoding consensus IN (IN_a), inactivated consensus IN (IN_in), inactivated consensus IN with mutations conferring resistance to elvitegravir (IN_in_e3), or empty vector. Splenocytes were stimulated *in vitro* with a Luc-derived peptide (LUC), and individual or pooled IN-derived peptides (Table 3) as described in Methods. IN-specific *in vitro* secretion of IFN-γ (**A**), IL-2 (**B**), and dual secretion of IFN-γ/IL-2 (**C**). Responses represent the average number of signal-forming units (sfu) per mln cells in two independent experiment runs, each done in duplicate,+SD.

All IN variants induced an equally good immune response in terms of IFN-γ, IL-2 and dual IFN-γ/IL-2 production by T-cells in response to *in vitro* stimulation with IN-derived peptides, as manifested by 500 to 1000 cells per mln splenocytes producing IFN-γ or IL-2, and up to 500 cells producing IFN-γ and IL-2 in all three groups (Table 3; p>0.1, Kruskal-Wallis test, except for IN_in_e3) (Fig. 5, and Fig. S2). IFN-γ and IL-2 were predominantly produced after stimulation of lymphocytes with peptides representing a cluster of human and murine CD4^+^ and CD8^+^ epitopes at aa 209-239, more precisely at aa 219-238 [10], [36], [51], [52], [53], [54] (peptides IN209 and MIN219, respectively, Table 3). IL-2 was also secreted after *in vitro* stimulation of splenocytes with peptides representing other known mouse epitopes (IN169; its shorter variant MIN169; a mixture of peptides MIN79; its IN_in_e3 derived variant MIN79e3; and MIN169; Table 3, Fig. 5). As could be expected, mouse T cells recognize neither the consensus IN-derived peptides corresponding to the known human CD8+ CTL epitopes of IN clade B (at aa 47–54, 66–74, 68–77, 84–94, 92–102, 143–151, and 151–158; www.hiv.lanl.gov and www.immuneepitope.org), nor their variants with elvitegravir-resistance mutations (Fig. 5). T cell responses were highly specific as they were seen only in mice immunized with IN DNA (p<0.05 compared to empty vector, Mann-Whitney test), whereas a T cell response against a peptide representing the CD8^+^ T cell epitope of luciferase (LUC) was seen in all mice (since Luc reporter gene was administered to all groups, including the controls; Fig. 5, Fig. S2).

**Table 3:** Peptides and peptide pools used in *in vitro* T-cell stimulation tests.

Abbreviated name ofthe peptide	Position in aa residues; positions of the mutatedaa are given in brackets	Amino acid sequence of the peptide;mutated aa are shown in bold
**Peptides derived from consensus integrase of HIV-1 FSU-A** *		
IN**66	66–98	THLEGKVIIVAVHVASGYIEAEVIPAETGQETA
IN169	169–196	AEHLKTAVQMAVFIHNFKRKGGIGGYSA
IN209	209–239	QTKELQKQIIKIQNFRVYYRDSRDPIWKGPA
**Pool_MIN** ** **(MIN79, MIN79e3, MIN169, MIN209, MIN219) contains** * **:**		
MIN79	79–98	VASGYIEAEVIPAETGQETA
MIN79e3	79–98 (92)	VASGYIEAEVIPA**Q**TGQETA
MIN169	169–190	AEHLKTAVQMAVFIHNFKRKGG
MIN209	209–228	QTKELQKQIIKIQNFRVYYR
MIN219	219–238	KIQNFRVYYRDSRDPIWKGP
**Pool_CTL (CIN47, CIN66, CIN68, CIN84, CIN92, CIN143, CIN151) contains** * **:**		
CIN47	47–54	GEAMHGQV
CIN66	66–74	THLEGKIII
CIN68	68–77	LEGKVIIVAV
CIN84	84–94	IEAEVIPAETG
CIN92	92–102	ETGQETAYFLL
CIN143	143–151	YNPQSQGVV
CIN151	151–158	VESMNKEL
**Pool_CTLe3 (CIN47e3, CIN66, CIN68, CIN84e3, CIN92e3, CIN143e3, CIN151e3) contains** *** **:**		
CIN47e3	47–54 (51)	GEAM**Y**GQV
CIN66	66–74	THLEGKIII
CIN68	68–77	LEGKVIIVAV
CIN84e3	84–94 (92)	IEAEVIPA**Q**TG
CIN92e3	92–102 (92)	**Q**TGQETAYFLL
CIN143e3	143–151 (147)	YNPQ**G**QGVV
CIN151e3	151–158 (157)	VESMNK**Q**L

* Choice of peptides done based on the epitopes mapped to these regions earlier [10], [36], [51], [52], [53], [54], [95]. Pool_CTL contains peptides representing human CTL epitopes mapped to the given regions.

** IN series includes peptides recognized by human T cells; and MIN, by T cells of H2-K^d^- restricted BALB/c mice.

*** Peptides of pool_CTL with mutations of resistance to elvitegravir where applicable.

**Figure 6:**
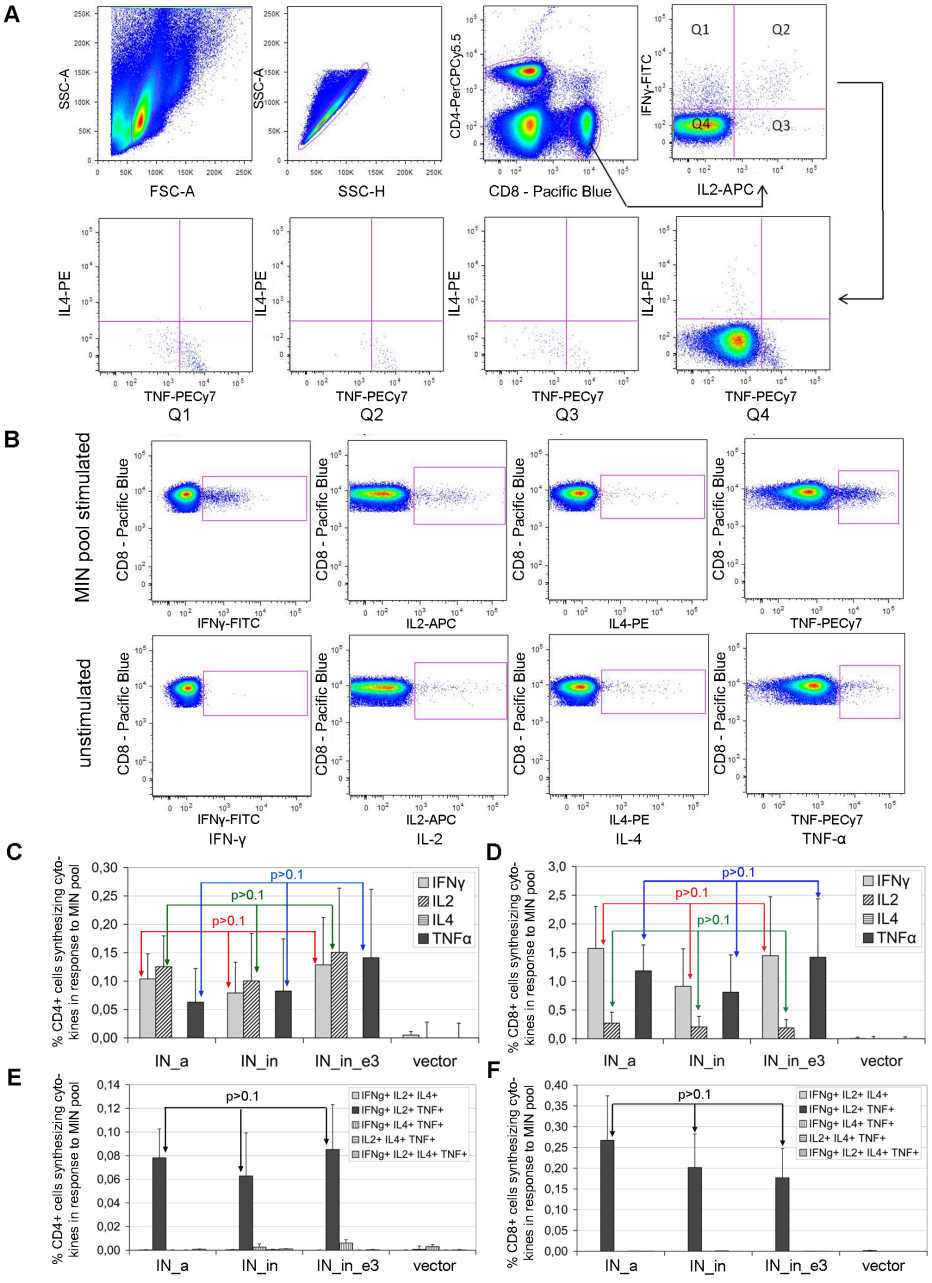
Multiparametric FACS assays of the multi-functional T-cell responses elicited by IN genes in BALB/c mice. Gating scheme used to identify CD4^+^ and CD8^+^ T cells. Side-scatter (SSC) parameters were used to identify single-cell events. CD4^+^ cells were defined; gating for the multifunctional CD8^+^ T-cells is shown in the lower panel. The same gating procedure was applied for CD4^+^ T-cells (**A**). Representation of a functional response to MIN peptide pool in the splenocytes of a mouse immunized with pVaxIN_in_e3 (upper row) relative to unstimulated splenocytes (lower row) (**B**). *In vitro* stimulation with the MIN-peptide pool (Table 1) resulting in the secretion of IFN-γ, IL-2, IL-4 and TNF-α (**C, D**) and multiple cytokine secretion (**E, F**): % responding cells of the total CD4^+^ positive (**C, E**), and of the total CD8^+^ positive cells (**D, F**). Boolean gate calculation platform was used to analyze the frequency of all possible combinations of IFNγ, tumor necrosis factor-α and interleukin-2 expression. Error bars represent SD.

The phenotype of responding cells was further evaluated by six-color flow cytometry assessing a surface expression of CD4^+^ or CD8^+^ and an intracellular expression of IFN-γ, IL-2, IL-4, and/or TNF-α. In this experiment, splenocytes were stimulated by a MIN peptide pool representing known CD4^+^ and CD8^+^ T cell epitopes of mice (Pool_MIN, Table 3), LUC peptide to control the response to Luc reporter, ConA as a positive control, or medium alone. Data from individual splenocytes collected by flow cytometry were subjected to the gating approach shown in Fig. 6A. A sample representative of cytokine expression by CD8^+^ T cells of IN_in_e3-immunized mice in response to *in vitro* stimulation with the MIN peptide pool, or medium is shown in Fig. 6B. No significant mouse-to-mouse difference in cytokine production was observed for unstimulated CD4^+^ or CD8^+^ cells or for cells stimulated with mitogen ConA (Fig. 6B, and data not shown). Mouse groups were thus similar with respect to the levels of unspecific reactivities and cell viability. As expected, the CD4^+^ and CD8^+^ T cell response to LUC peptide was similar in all groups, including the control group which received Luc gene together with the empty vector (Fig. S3). No difference in anti-reporter immunity between the groups indicated the uniformity of immunization (plasmids being equally well delivered into all groups). This created an ideal set-up for an accurate comparison of specific responses to the three IN genes.

CD4^+^ and CD8^+^ responses against the peptide pool representing known mouse CD4^+^ and CD8^+^ T cell epitopes (MIN) was detected in all IN gene recipients (Fig. 6B). The percentage of CD4+ and CD8+ T cells expressing single and multiple cytokines determined after application of the Boolean gating strategy is given in Fig. 6C–F. Up to 0.14% of the total CD4^+^ T cells were positive for IFN-γ, IL-2, and/or TNF-α (Fig. 6C). CD8^+^ T cells responded mainly by secretion of IFN-γ and TNF-α, with 0.6 to 1.6% of cells positive for each of the cytokines. IL-2 was produced by about 0.2% of the CD8+ T cells (in all IN-immunized groups; Fig. 6D). None of the IN gene variants induced any detectable IL-4 production (Fig. 6 C, D; Fig. S4). The strongest single-cytokine response was elicited in the IN_a gene immunized mice; % of single-cytokine positive CD4^+^ and CD8^+^ T cells in this group significantly exceeded the respective numbers in the control animals (p<0.03, Mann-Whitney U-test). Inactivated consensus IN and its variant with elvitegravir resistance mutations demonstrated somewhat higher IFN-γ, IL-2 and TNF-α responses than the control mice, but the difference did not reach the level of significance (p>0.05; Fig. 6C, D). There were no difference in specific cytokine secretion between groups of mice immunized with different IN genes (p>0.1, Kruskal-Wallis test; Fig. 6 C, D).

Importantly, immunization with all three IN genes elicited a significant number of IN-specific CD4^+^ and CD8^+^ T cells which simultaneously produced IFN-γ, IL-2 and TNF-α (Fig. 6 E, F). The number of CD4^+^ and CD8^+^ T cells triple-positive for IFN-γ, IL-2 and TNF-α in mice receiving the IN genes was equally high in all three groups (p>0.1, Kruskal-Wallis test; Fig. 6 E, F), and significantly exceeded that in the control vector-immunized mice (p<0.05 in pair-wise group comparisons by Mann-Whitney test; Fig. S4 A, B; Fig. 6 E, F).

### IN Gene Immunization Induces Specific Antibody Response

**Figure 7:**
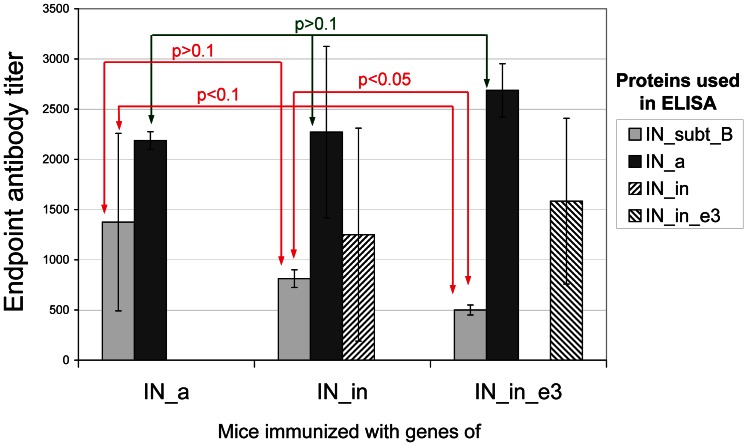
Immunization with the IN gene variants induces cross-reactive IN-specific IgGs. End-point titers of the integrase-specific IgG antibodies in the sera of BALB/c mice immunized with the genes encoding consensus HIV-1 FSU-A integrase (IN_a), consensus IN inactivated by D64V mutation (IN_in), and inactivated consensus integrase carrying mutations conferring resistance to elvitegravir (IN_in_e3). Data represent a mean ±SD for the end-point titer of antibodies against the consensus IN variants and IN of HIV-1 HXB2 strain (IN_subt_B) for four mice per group in two independent immunization runs. Cut-offs were set against the control mice immunized with the empty vector (see Methods for details).

Sera from BALB/c mice immunized with IN gene variants collected after the completion of immunization was subjected to indirect ELISA on plates coated with the IN variants. IN gene immunization was found to induce IN-specific IgG in the average titers from 500 to 2500 (Fig. 7). IN_a was equally well recognized in all three groups (p>0.1, Kruskal-Wallis test, Fig. 7), IgG titers varied from 200 to 3000. Interestingly, active consensus integrase was better recognized by the sera of mice immunized with the most divergent IN variant IN_in_e3: in this group the individual anti_IN_a titers reached 3000. Mice receiving IN gene variant IN_in_e3 demonstrated the lowest anti-IN clade B antibody titers (500; Fig. 7). This contrasted with their high ability to recognize the consensus active integrase of FSU-A strain. Titer of antibodies against IN of clade B in mice immunized with IN_in_e3 was lower than in mice receiving IN_in gene (p<0.05, Fig. 7). The overall antibody recognition of IN_clade B was weak with the average antibody titers less than 1500 (Fig. 7). Recognition of mutant FSU-A integrases IN_in and IN_in_e3 was tested only in mouse groups immunized with respective variants (the average titer from 1000 to 1500; Fig. 7).

### In vivo Assessment of the Effector Capacity of Anti-integrase Immune Response

**Figure 8:**
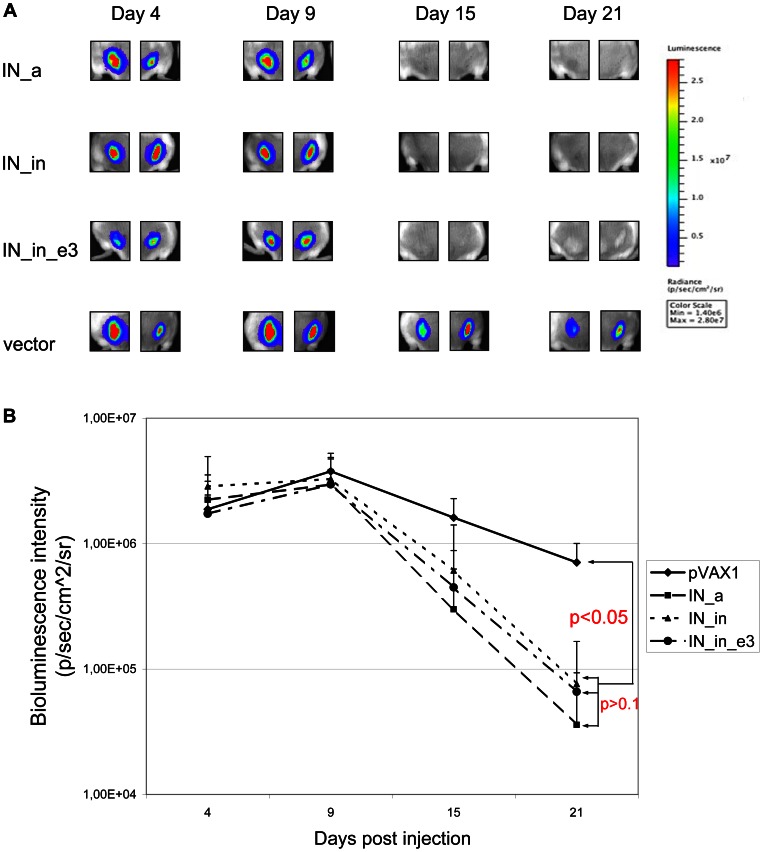
Dynamics of bioluminescence at the sites of the IN gene and luciferase reporter gene co-administration. *In vivo* monitoring of luciferase activity at days 4, 9, 15 and 21 after the administration of plasmids encoding the consensus IN (IN_a), inactivated consensus IN (IN_in), inactivated consensus IN with elvitegravir resistance mutations (IN_in_e3), or empty vector pVax1, each mixed with Luc reporter gene (1∶1). Images demonstrate two representative injection sites per group followed throughout the immunization. The scale to the right represents the strength of luminescent signal in pixels/sec/cmˆ2/sr (**A**). Kinetics of the luciferase expression over time (four mice in each group; two independent experiments) (**B**).

**Figure 9:**
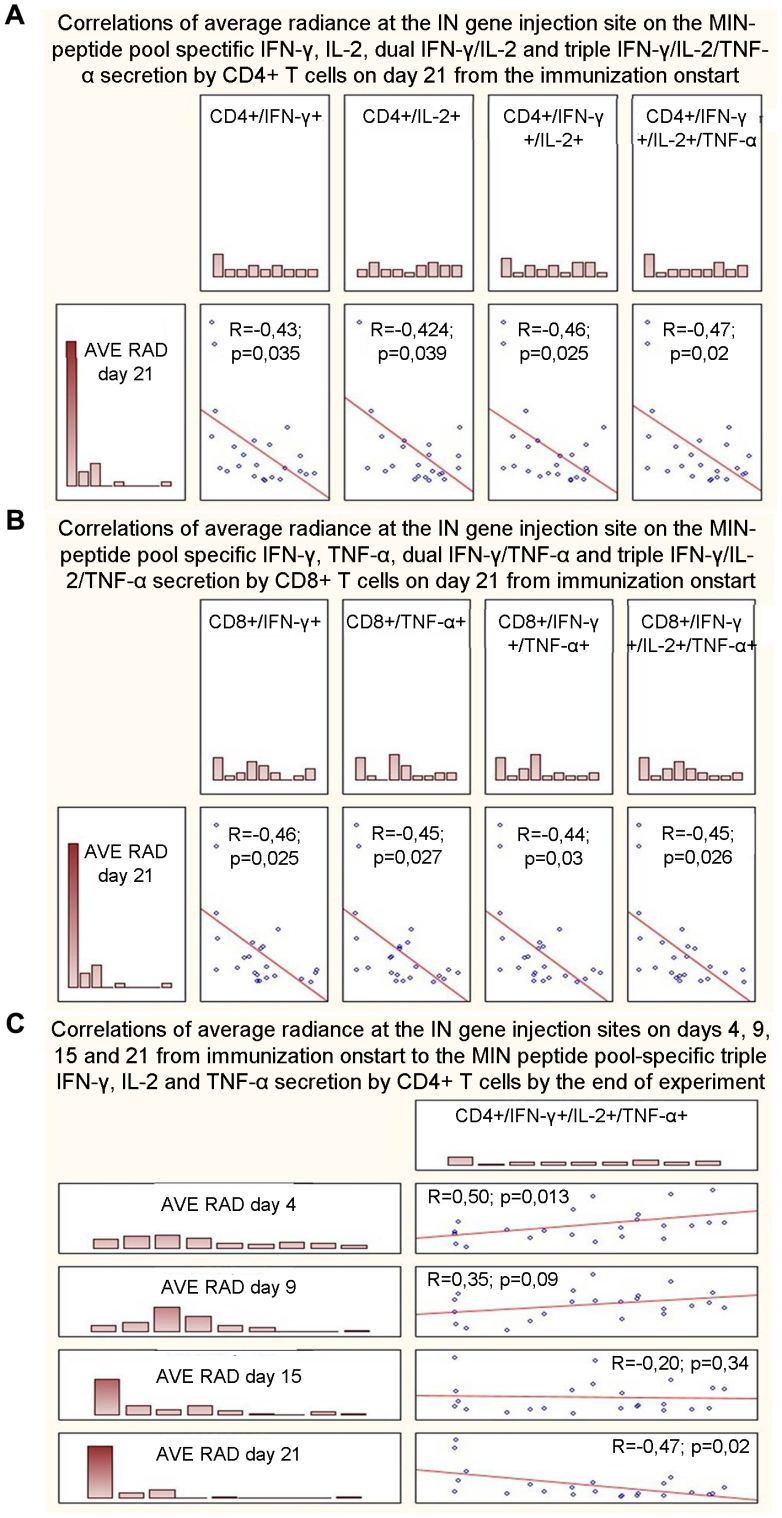
Average radiance at the sites of the IN/Luc-reporter genes co-injection correlates to IN-specific cytokine response. Inverse correlation of the bioluminescence represented by the average radiance (BLI) at the injection site on day 21 after the immunization to: % CD4+T cells secreting IFN-γ, IL-2, IFN-γ/IL-2, and IFN-γ/IL-2/TNF-α (**A**); % CD8+T cells secreting IFN-γ, TNF-α, IFN-γ/TNF-α and IFN-γ/IL-2/TNF-α (**B**). Correlations of BLI on days 4, 9, 15 and 21 to the triple IFN-γ/IL-2/TNF-α secretion by CD4+ T cells by day 23 (**C**). Results of the BLI and FACS analysis of the data collected in two independent experiments (2 times × 4 mice in each group) were analyzed by the Spearman rank-order test (Statistica AXA 10).

Next, we investigated whether the immunization with IN gene variants influences the *in vivo* expression of the transfected genes. For this, we followed the expression at the sites of immunization of the reporter gene encoding firefly luciferase (Luc) co-delivered as a 1∶1 mixture with IN gene variants. By day 21, the expression of luciferase in mice receiving Luc and IN genes had significantly decreased, while little change was registered in mice receiving Luc gene together with an empty vector (p<0.00001; Fig. 8A, B). The decrease in the luminescent signal emitted from the sites of injection of the integrase and the reporter gene was similar for IN_a, IN_in and IN_in_e3 groups starting from day 9 and up to day 21 (p>0,1 in all these time points by both Kruskal-Wallis and Mann-Whitney tests; Fig. 8B). Luminescence on day 21 inversely correlated with the end-point (day 23) IFN-γ, IL-2 and dual IFN-γ/IL-2 production by CD4+ and with IFN-γ, TNF-α and dual IFN-γ/TNF-α production by CD8+ T-cells (all ps <0,05; Fig. 9A, B). Equally strong inverse correlations were found between the end-point luminescence and the magnitude of integrase-specific triple cytokine response of CD4^+^ and of CD8+ T cells (Fig. 9A, B). Interestingly, luminescence at the early time points, as day 4, directly correlated with the end-point immune response (Fig. 9C). The latter indicated that the magnitude of T cell responses is predetermined by the efficacy of gene transfer and initial expression, while the induction of the integrase-specific multicytokine response of CD4+ and CD8+ T cells leads to the loss of luciferase reporter activity at the immunization sites.

## Discussion

HIV-1 integrase inserts the proviral DNA into the host genome securing the life-long viral infection [27]; [34]; [35]. Alongside with reverse transcriptase and protease, it plays a key role in HIV-1 drug resistance [55]; [56]. The newest HIV-1 inhibitors targeting integrase have a low genetic barrier to resistance (one to two mutations are sufficient to make the enzyme drug-insensitive) [57] and it is only a matter of time before the resistance to integrase inhibitors reaches alarming levels [3]. Integrase induces a strong immunodominant CTL response [58], [59], [36] but despite the immune pressure, remains highly conserved in order to preserve the activity (integrase activity depends on the full preservation of up to two-thirds of the protein; [60]). High conservation, immunogenicity and absence of counterparts in the cellular machinery positioned integrase as an ideal target for exerting bottle-neck immune pressure on the virus.

We hypothesized that an effective immune response against HIV integrase including its drug-resistant forms may interfere with the viral evolution towards drug-resistant phenotype(s). This prompted us to design a series of novel integrase-based gene immunogens. Specifically, we constructed the consensus integrase of HIV-1 FSU-A based on 40 complete FSU-A *pol* gene sequences originating from the territory of the former Soviet Union. The amino acid sequences of FSU-A integrases appeared to be very homogeneous; 80% of the amino acid consensus was fully conserved. This consensus sequence was further modified to inactivate the enzyme, make it stable, and ensure its high-level expression. For this, the consensus IN gene was codon-optimized and modified toward stability. Viral IN has the N-terminal Phe residue, which makes it a substrate of the N-end rule pathway [46]. The N-terminal Phe was substituted with a dipeptide Met-Gly, since the N-terminal Met residue aids to the protein stability [45], [48], [61]. The Met-Gly-extension did not affect protein structure and folding as indicated by the consensus FSU-A enzymatic activity which exceeded the activity of the viral HIV-1 HXB2 integrase. To make the immunogen safe, the consensus IN was inactivated (IN_in) by substituting Asp64 in the IN catalytic triad for Val, which prevents strand transfer activity [62], [63].

The inactivated IN was provided with mutations conferring resistance to elvitegravir, a novel strand transfer inhibitor currently in Phase III clinical trials [64]. For HIV-1 clade A, the main mutations of elvitegravir resistance are H51Y, E92Q, S147G, along with E157Q and a secondary nonpolymorphic mutation, K160Q, highly infrequent in integrase inhibitor-naive patients [49], [50]; introduction of these mutations generated IN derivative IN_in_e3.

Activity tests done on D64V-IN variants produced in *E. coli* demonstrated that they had no strand-transfer activity, and their genes are, therefore, safe to use in immunization. All three integrase variants (IN_a, IN_in, IN_in_e3) were highly expressed in human and murine cells. The level of eukaryotic expression reached 700 pg per cell, exceeding the levels observed for the virus-derived HIV-1 enzyme genes by almost 50-fold [65]. None of the mutations (inactivating or conferring resistance) had any effect on the level of IN expression. Thus, the humanized IN genes met all criteria set for the effective gene immunogens.

This was confirmed by the results of the IN gene immunization of BALB/c mice. All three IN genes were strongly immunogenic for mouse T cells. CD8+ and CD4+ T cell responses were mainly directed against a cluster of epitopes at aa 209–239 of IN (peptides IN209, MIN219; Table 3). IFN-γ/IL-2 response of murine PBMC against this cluster was registered already on day 15 after immunization (Fig. S2). By day 27, T cell responses of splenocytes to stimulation with IN209 and MIN219 had significantly expanded (Fig. 5). IN aa 209–239 of consensus HIV-1 clade A appeared to contain a murine T cell epitope(s) (or epitope cluster). A strong T cell response against this region induced by all IN gene variants suggested its use as a lead-epitope to monitor integrase-specific T cell responses. Recognition of other peptides representing human and mouse T cell epitopes localized at aa 66–98 and 169–190 (IN69, MIN79, and IN/MIN169, respectively; Table 3) was weak and occurred mainly in the form of IL-2 production (Fig. 5).

T cell stimulation by IN-derived peptides was further analyzed by multiparametric FACS. In all groups receiving IN genes, stimulation by the pool of peptides representing mouse CD4+ and CD8+ T cell epitopes (pool_MIN; Table 3) triggered production of IFN-γ, IL-2, and/or TNF-α by 0.08 to 0.14% CD4^+^ cells, of IFN-γ or TNF-α by 0.8 to 1.6% CD8+, and of IL-2 by 0.2% CD8+ T cells (Fig 6). None of the stimulated T cells produced IL-4. IFN-γ is the most commonly measured cytokine associated with protection against viral infections. Thus, all three synthetic IN genes behaved as effective gene immunogens able to induce potent Th1-type responses in both CD8+ and CD4+ T cells. Secretion of both IFN-γ and TNF-α by effector CD8+ T cells is critically important for protection against viral infections [66]. IL-2 supports the secondary expansion of memory CD8+ T cells and generation of the long-term protective immunity [67], [68]. Generation of all three cytokines is considered to be a prerequisite for an efficient antiviral immunization.

Production of cytokines is hierarchical in character: most of the epitope-specific CTLs produce IFN-γ, some, IFN-γ+TNF-α+, and still a smaller subset, referred to as “polyfunctional”, all three cytokines [69], [70]. Polyfunctional T cells have been associated with an effective control of intracellular infections, specifically of viral replication, and with strong protection in vaccination [71], [72], [73], [74], [75], [76]. In HIV-1 infection, multiple cytokine secretion by lymphoid cells has been associated with T cell suppressor activity, superior control of HIV-1 replication, and long-term non-progression to AIDS [71], [77], [78], [79]. In mice immunized with IN gene variants, all IL-2 positive CD8+ T cells stimulated with IN peptides secreted IFN-γ and TNF-α; 0.2% of CD8+ T cells co-expressed IFN-γ, IL-2 and TNF-α and thus belonged to the polyfunctional Tc1 phenotype. The majority of CD4+ T cells also co-expressed either two (IFN-γ and TNF-α, 0.02%) or all three cytokines (IL-2, IFN-γ and TNF-α; 0.06%) and thus belonged to the polyfunctional Tc1 phenotype. Co-expression of TNF-α and IFN-γ indicated that these IN-specific CD4+ T cells were the effectors acting through TRAIL-mediated apoptosis [80], [81], while co-secretion of IFN-γ, TNF-α and IL-2 identified the population of effector CD4+ T cells capable of perforin-mediated target cell killing [82]. The perforin- and cytotoxic cytokines/TRAIL-based killing account for the bulk of lytic activities of CD4+ T cells [80], [81]. Immunization with IN gene variants was apparently able to trigger at least one of the effector mechanisms.

Furthermore, IN gene immunization generated integrase-specific antibodies which recognized both the consensus FSU-A integrase and a clade B (HXB2) integrase with similar end-point titers. Thus, IN gene variants could induce antibodies against epitopes common for integrases of clade A and B.

Finally, we evaluated the capacity of the elicited anti-IN immune response to eliminate the transfected expressing cells from the immunization sites. This was done by assessing the level of expression in the injection sites of the reporter gene of firefly luciferase, co-delivered with the IN gene variants [83]. As we have recently shown, co-injection of Luc reporter gene with a potent gene immunogen results in a rapid loss of the *in vivo* reporter activity (decrease of bioluminescence) [83]. Here, co-delivery of Luc and IN genes led to a significant, 10- to 15-fold decrease in the total photon flux from the site of immunization three weeks post immunization. We found inverse correlations of luminescence with IFN-γ/TNF-α and IFN-γ/IL-2/TNF-α expression by CD8^+^ and with dual IFN-γ/IL-2 and triple IFN-γ/IL-2/TNF-α expression by CD4^+^ T cells (Fig. 9A, B). Correlations of luminescence with IFN-γ/TNF-α production by CD4+, and with IFN-γ/IL-2 production by CD8+ T cells did not reach the level of significance indicating that to affect the luminescence, CD4+ T cells depended on IL-2, and CD8+ T cells, on TNF-α, each featuring the respective effector (lytic) T cells. This supported the concept of luminescent “fading” being due to the T-cell mediated clearance of the expressing cells from immunization sites. Further, this indicates the role in clearance of immunogen/reporter expressing cells of the lytic CD4+ Th1 cells. Lytic CD4^+^ T cell clones can suppress replication of HIV and SIV in both CD4^+^ T cells and macrophages [84], [85]. Induction of such effectors would offer a possibility to attack virus-infected cells via the MHC class II pathway (less prone to escape mutations than the MHC class I pathway for CD8^+^ T cells) and also to recognize and kill macrophages that serve as a long-lived reservoir for HIV-1. Both capacities would clearly benefit a multi-component/multi-gene HIV-1 vaccine.

### Conclusions

We have shown that the consensus genes encoding inactivated HIV clade A integrase and its analog with primary elvitegravir resistance mutations are immunogenic for both T and B cells. We have described T cell immune response against the consensus integrase and found that it is executed by the polyfunctional CD8^+^ and CD4^+^ T cells co-secreting IFN-γ, IL-2 and TNF-α. We have characterized the functionality of this immune response in the *in vivo* tests as the capacity to reduce local expression of the reporter gene co-delivered with the IN gene immunogens. The latter correlated with the induction of IN-specific response of polyfunctional CD8^+^ and CD4^+^ T cells with a lytic phenotype, and was, therefore, interpreted as the immune-mediated extermination of the expressing cells. Generation of such polyfunctional CD4+ and CD8+ T cell response is highly desirable for an effective HIV-1 vaccine as it would offer a possibility to attack virus-infected cells via both MHC class I and MHC class II pathways. Generation of such polyfunctional T cells is highly desirable for an effective HIV-1 vaccine [86]. Several recent HIV-1 multigene vaccine trials have included the IN gene [39], [40], [87] which supports its perspectivity for immune therapy of HIV/AIDS, specifically, the immune prevention of drug-resistance. Our consensus HIV-1 clade A immunogens would be specifically adapted to hinder epidemics caused by HIV-1 strains with low genetic diversity as in the Russian Federation [41], [42], [43].

## Methods

### Ethics Statement

All experiments were approved by the Northern Stockholm’s Unit of the Ethics of Animal Research on 2010-08-26, ethical permission N197/10 “Evaluation of the new generation of vaccines against highly dangerous infectious diseases and cancer”. The experiments conveyed under this ethical permission aimed to develop new vaccines and new vaccination strategies against cancer and serious viral infections as HIV, and to advance new treatment protocol for further clinical applications. Vaccine candidates to test under the application included naked DNA vaccines, proteins, peptides and viral vectors administered with or without adjuvants. Immunization were allowed by intramuscular, subcutaneous and intradermal injections, inoculations with Biojector with or without electroporation, and nasal immunization with drops. All injections, biojections and electroporation were made under the inhalation anesthesia with a mixture of air and 1.5 to 3% isofluorane.

All procedures were evaluated as having low to average degree of difficulty. Methods of immunization and follow-up were not painful; earlier done experiments had shown no effect of the procedures on the gain of the weight, water or food consumption or overall mouse behavior. Possible mouse discomfort under immunization monitoring and sample collection was relieved by the inhalation anesthesia. Animals were sacrificed by cervical dislocation.

In animal experiments conveyed in the present study, BALB/c (H2-Dd) mice (females, 8 week old) were purchased from Charles River Laboratories (Sandhofer, Germany) and housed at the Astrid Fagrius Laboratory, Karolinska Institute, Stockholm, Sweden. Mice were contained in the environment-enriched cages, 5–8 animals per cage. Food and water were supplied *ad librum*. Animals were regularly controlled for the food and water intake, weight development, skin and fur changes and microscopical alterations at the site of immunization. Gene injections were made intradermally with ≤30 G needles with volume never exceeding 20 microliters. To screen the immune response, mice were bled from the tail vein two and four weeks after immunization. Gene expression was assessed with the help of reporters using *in vivo* imaging technique (IVIS). IVIS monitoring was performed in the thermoregulated dark chamber for ten to sixty seconds. Prior to intradermal injection, electroporation, bleeding, and during live imaging, the mice were anesthetized with a mixture of air and isofluorane, 2–2.5% during induction and 1.5–3% thereafter. Mixture was delivered in the inhalation chamber or via nasal masks (Baxter Medical AB, Kista, Sweden).

### Synthetic Integrase Genes

Full-length HIV-1 clade A integrase sequences from treatment naïve patients isolated in the territory of the former Soviet Union (n = 34; Belarus, Estonia, Georgia, Russia, Ukraine, and Uzbekistan) were selected ([44]; and V. Lukashov, unpublished). Integrase consensus was created using BioEdit software (Ibis Biosceinces, Carlsbad, CA). A humanized synthetic gene encoding the respective amino acid sequence (IN_a) was designed using the web service utility at http://genomes.urv.es/OPTIMIZER [88] and the on-line customer portal at http://www.invitrogen.com. The N-terminal Phe residue of IN was exchanged for Met-Gly. This together with the insertion of an ATT triplet upstream of the AUG codon introduced the consensus Kozak’s sequence ANNATGG. The resulting mRNA was checked for the absence of undesirable folding (UNAFold at http://mfold.rna.albany.edu/, and OPTIMIZER at http://genomes.urv.es/OPTIMIZER/). For cloning into pVax1, the synthetic DNA duplex was flanked with extra 5′- and 3′-terminal sequences: GGATCC prior to the ATT-ATG-GGC sequence at the 5′-terminus introducing *BamHI*, and GAATTC following TGA stop-codon at the 3′-terminus introducing *EcoRI* restriction sites. The consensus coding sequence was synthesized by Evrogen (Moscow, Russia). Deoxyribopolynucleotides encoding inactivated IN (IN_in: D64V) and inactivated elvitegravir-resistant IN (IN_in_e3: H51Y, D64V, E92Q, S147G, E157Q, K160Q) were obtained by site-directed mutagenesis of IN_a gene.

### Oligonucleotides, Peptides, and Proteins

Oligonucleotides were synthesized using an Applied Biosystems 380B DNA synthesizer and purified by electrophoresis in a 20% denaturing polyacrylamide gel. To choose peptides for IN-specific immune assays, sequences of consensus FSU-A and clade B integrases were aligned, and regions in FSU-A IN were defined which were homologous to the known epitopes of integrase of HIV-1 clades A, B, and C [10], [36], [51], [52], [53], [54], [44], [89]. Respective synthetic peptides (Table 3) were purchased from GL Biochem Ltd (Shanghai, China). Control peptide LUC (GFQSMYTFV; GL Biochem Ltd) represented a H2-Kd restricted CTL epitope of firefly luciferase [90]. Integrase of HIV-1 subtype B bearing 6His tail was expressed in *E. coli* and purified by affinity chromatography as described previously [91].

### Anti-integrase Antibodies

Chinchilla grey rabbits (2.5–2.8 kg, 8–10 weeks old; Federal State Enterprise “Manikhino” Istrinsky area, Moscow region, Russia) were primed by subcutaneous injection of IN of HIV-1 HXB2 (30 µg in 50 µl PBS mixed with the complete Freund adjuvant 1∶1 v/v) at days 1 and 6, and then boosted three times with one-month intervals with 15 µg of IN in 200 µl PBS mixed with the incomplete Freund adjuvant (1∶1 v/v). Blood collected two weeks post the last boost had an end-point anti-IN antibody titer of >10^5^ in indirect ELISA. ELISA was performed on IN HXB2-coated plates (MaxiSorb, Nunc) with detection using secondary horseradish peroxidase (HRP) conjugated anti-rabbit antibodies (DAKO, Denmark) as described below for the mouse sera.

### Cloning of IN Genes for eu- and Prokaryotic Expression

IN_a, IN_in, IN_a_e3 and IN_in_e3 coding sequences were cloned into a pVax1 vector (Invitrogen) using *BamHI* and *EcoRI* restriction sites generating plasmids pVax1IN_a, pVax1IN_in and pVax1IN_in_e3, respectively. To create prokaryotic expression vectors, IN_a, IN_in, and IN_in_e3 coding sequences were PCR-amplified from the pVax1-based plasmids using Pfu polymerase (Promega) and primers: forward 5′–TGACCATATGGGCTTCCTGGAGGG–3′ and reverse 5′–TGACGGATCCTAGTCCTCATCCTGTCTGCTG–3′ containing *NdeI* and *EcoRI* restriction sites. PCR products were digested with *NdeI* and *EcoRI* (Fermentas, Vilnius, Lithuania) and ligated into the *NdeI/EcoRI*-cleaved plasmid pET15b-IN (a kind gift of Prof. J-F Mouscadet, Cachane, France) in frame with the codons for the N-terminal 6His tag in replacement for the coding sequence of HIV-1 HXB2. Ligation mixtures were transformed into competent OneShotTop10 *E.coli* cells (Invitrogen, Sweden) by heat shock. Clones obtained on the selective media were screened by PCR using cloning primers. All pVax1- and pET-based plasmids were purified using a miniprep kit (Qiagen, Sweden) and sequenced (Eurofins MWG Operon, Germany).

### Prokaryotic Expression and Purification of Integrases

Integrase variants of HIV-1 subtype A bearing a 6His tail were expressed in *E. coli* BL21(DE3) host strain (Novagen®, Merck Millipore, Darmstadt, Germany and Billerica, MA, USA) with pRARE plasmid from Rosetta (DE3) strain (Novagen). Protein expression was induced by adding IPTG, and integrases were purified by affinity chromatography, as described previously [91]. Fractions were analyzed by electrophoresis in 12% SDS-PAGE with subsequent Western blot using polyclonal anti-IN rabbit sera. Quantitative image analysis of the Coomassie-stained gels with Image-QuantTM 4.1 software (Amersham Biosciences Corp, Piscataway, NJ) revealed each IN preparation to be at least 80% pure. Protein concentration in purified IN preparations was determined by micro-Bradford assay (BioRad, USA). Fractions were aliquoted and frozen at −80°C.

### Integrase Activity Assays

#### DNA duplexes for assessing integrase activity

Integrase activities were assessed using synthetic DNA duplexes (Table 1). DNA duplex U5 consisting of the oligonucleotides U5B and U5A, which mimicked the end of HIV-1 U5 LTR, served as a substrate for 3′ processing activity. Duplex U5-2, formed by U5B-2 and U5A, was used as a substrate for strand transfer and duplex Ran formed by oligonucleotides RanB and RanA, to verify the specificity of 3′-processing. To measure integrase catalytic activities, the oligonucleotides U5B, U5B-2, and RanB (10 pmol each) were labeled using T4 polynucleotide kinase and 50 µCi of [γ-^32^P]ATP (3000 Ci/mmol). After 1 hour of incubation at 37°C, EDTA was added to the final concentration of 50 mM, and the reaction mixture was heated for 5 minutes at 65°C to inactivate the kinase. Labeled oligonucleotides were supplemented with equimolar amounts of unlabeled complementary oligonucleotides and annealed by first heating for 3 minutes at 90°C and then cooling slowly to room temperature. Resulting duplexes were purified using Micro Bio-Spin columns P-6 (Bio-Rad, Berkeley, CA).

#### 3′-end processing and strand transfer reactions

All assays were carried out as described previously [92]. DNA duplexes (10 nM) were incubated for 2 hours with 100 nM IN in 20 µl of the buffer containing 20 mM Hepes, pH 7.2, 7.5 mM MgCl_2_, and 1 mM DTT, at 37°C. DNA fragments were precipitated with ethanol and separated in denaturing 20% polyacrylamide gels. Gels were analyzed on a Storm 840TM PhosphorImager (Molecular Dynamics, Sunnyvale, CA, USA) and quantified with Image-QuantTM 4.1 software (Amersham Biosciences Corp, Piscataway, NJ). Integrase activity was defined as percent substrate converted to a product; activities of IN variants were quantified relative to IN_a values. Each experiment was repeated at least three times with convergent results.

### Eukaryotic Expression of Integrases

HEK293, HeLa and NIH3T3 cells (ATCC, Manassas, VA) were cultured in the Dulbecco’s modified Eagle’s medium (DMEM) supplemented with 10% fetal bovine serum (FBS) at 37°C in 5% CO_2_ humidified atmosphere. Cells were transfected with pVaxIN_a, pVaxIN_in, pVaxIN_in_e3, or empty vector pVax1 using Lipofectamine LTX (Invitrogen Corporation, Carlsbad, CA). At hour 48 post-transfection, cells were harvested, lysed and analyzed by electrophoresis in 12% SDS-PAAG with subsequent Western blotting (Bio-Rad, Hercules, CA, USA), using for staining polyclonal anti-IN rabbit sera (1∶10000). Binding was visualized by secondary HRP-conjugated anti-rabbit antibody (DAKO; 1∶5000). The membrane was developed using the ECL plus western blotting detection system (GE Healthcare Amersham Biosciences, USA). To normalize for the total protein content, membranes were stripped according to the ECL protocol and re-stained with monoclonal mouse anti-actin antibody (Sigma-Aldrich, St. Louis, MO, USA; 1∶5000), followed by the HRP-conjugated anti-mouse antibody (DAKO; 1∶5000). Films were scanned and the relative intensity of the bands was estimated using ImageJ software (http://rsbweb.nih.gov/ij/). To assess the level of IN expression per cell, the percent of cells expressing IN was estimated from the efficacy of transfection established in a control co-transfection with a reporter GFP plasmid; % transfection gave the number of cells expressing IN among 5000 cells resolved by PAGE and Western blotting in one PAAG well. Calibration samples of recombinant IN in a range from 0.1 to 10 ng were resolved on the same gel. IN protein content in a lysate was quantified by plotting the intensity of the respective IN band on the film (grey units; Image J) against the IN calibration curve; IN content per cell was calculated by dividing this value by the number of expressing cells.

### DNA Immunization of Mice

BALB/c (H2-Dd) mice (females, 8 week old) were purchased from Charles River Laboratories (Sandhofer, Germany) and housed at the animal facility of the Karolinska Institute, Stockholm, Sweden. Groups of mice (4 per group) were immunized subcutaneously with pVaxIN_a, pVaxIN_in, pVaxIN_in_e3, or pVax1 (total of 20 µg in 20 µl PBS each) mixed with an equal amount of pVaxLuc reporter. Plasmids were delivered as two intradermal injections with a 29G insulin-grade syringe (Micro-Fine U-100; BD Consumer Healthcare, Franklin Lakes, NJ) on the lower back to the left and to the right of the base of the tail. Immediately after, a needle array electrode (1.5 × 4 mm gaps; Cellectis, Romainville, France) was placed over the injection site and voltage was applied using DermaVax electroporator (Cellectis) in a regimen optimal for small rodents [93].

On days 4, 9, 15 and 21 after the injection, mice were subjected to *in vivo* imaging of the reporter expression. At day 15, the mice were bled, and at day 22, bled and sacrificed, and spleens were collected. Prior to intradermal injection, electroporation, bleeding, and during live imaging, the mice were anesthetized with 2–2.5% isoflurane/air delivered in the inhalation chamber or via nasal masks (Baxter Medical AB, Kista, Sweden). All experiments were approved by the Swedish National Board for Laboratory Animals, ethical permission N197/10.

### In vivo Imaging of Reporter Expression after DNA Vaccination

To monitor luciferase expression *in vivo*, mice were injected i.p. with 15 mg/ml solution (100 µL per 10 g body weight) of D-luciferin potassium salt (Caliper Life Science, formerly Xenogen, Alameda, CA) in PBS; and let to move freely for 5 minutes. After that, mice were anesthetized for 5 min with 2–2.5% isoflurane in the inhalation chamber, and transferred into the *in vivo* imager (IVIS200, Caliper Life Science). Assessment of photonic emissions (photons/s/cm^2^) was performed for 1 minute. Luminescent and photographic images were captured by an in-built CCD camera and overlayed using Living Image software. A square-shaped frame was selected that engulfed each of the photon-emitting areas registered throughout the experiment cross groups and time-points (normally 10×10 mm). The frame was applied to all images in the series, and photons emitted from this area per minute were acquired as radiance per area (RAD, photons/s/cm^2^) using Living Image software version 2.50.1 (Caliper Life Science). Bioluminescence at one time-point was presented as an average of two sites in one mouse and as an average of all sites in a group.

### Assays of Antibody Response

Maxisorb 96-well microtiter plates (Nunc Maxisorp, Denmark) were coated with an IN protein variant in PBS at 0.3****µg/ml and incubated overnight at 6–8°C. Plates were washed six times with PBS containing 0.05% Tween-20. Individual mouse sera diluted step-wise from 1∶100 in HIV-Scan Buffer (HSB; 2% normal goat serum, 0,5% BSA, 0, 05% Tween-20, 0,01% sodium merthiolate) were applied and incubated overnight at 6–8°C. Plates were washed as above and HRP-conjugated goat anti-mouse IgG antibody (Sigma) diluted in HSB was applied and incubated for 1.5 hours at 37°C. Plates were washed as above and developed with 3,3′,5,5′-tetramethylbenzidine solution (TMB; Medico-Diagnostic Laboratory, Moscow, Russia). The reaction was stopped by 50 µl 2.5M sulfuric acid, and optical density (OD) was measured at a dual wavelength of 450–620 nm. The cut-off for specific anti-IN antibody response at each time-point was set to the mean OD-values demonstrated by the sera of the vector-immunized mice at this time-point +3 SD. For positive sera showing OD values exceeding the cut-off, end-point dilution titers were established from the titration curves.

### Assays of T-cell Responses

Blood samples collected on day 15 were pooled group-wise and peripheral blood mononuclear cells (PBMC) were purified by gradient centrifugation in Ficoll-Plaque Plus (GE Healthcare Amersham Biosciences, USA) as described [94]. Individual mouse spleens collected in day 21 were homogenized to obtain splenocytes. Single-cell spleen suspensions were treated with Red Blood Cell lysing buffer and re-suspended in RPMI supplemented with 2 mM L-glutamine, 2 mM Penicillin-Streptomycin (all from Sigma-Aldrich, St. Louis, MO) and 10% FBS (Gibco, Invitrogen, Carlsbad, California) (complete media).

#### Fluorospot assay

Fluorospot was performed on pooled PBMC or individual mouse splenocytes using an IFN-γ/IL-2 Fluorospot kit (Mabtech, Stockholm, Sweden) as described by the manufacturer. In brief, Fluorospot plates were treated with 35% ethanol, washed and coated with a mixture of monoclonal antibodies to IFN-γ and IL-2. 250,000 cells were added per well and stimulated with peptides (single, or pooled in equimolar amounts at a total concentration of 10 µg/ml), recombinant IN (6 and 12 µg/ml), medium alone, and Concanavalin A (Con A, 5 µg/ml) as a positive control. Plates were developed using specific monoclonal detection antibodies and fluorophore-conjugated secondary reagents. Finally plates were treated with a Fluorescence enhancer (Mabtech) to optimize detection and then dried. The number of cytokine-producing spot-forming cells (SFC) per million was evaluated using the AID iSpot FluoroSpot Reader System (AID GmbH, Strassberg, Germany). A net SFC/10^6^ cells in response to each antigen was calculated by subtracting the background response detected in the medium alone. The response to an antigen was considered specific if it exceeded the mean net response to the antigen in the empty vector-immunized mice +3SD.

#### Intracellular cytokine staining (ICCS)

All reagents used in ICCS were from BD Biosciences (Franklin Lakes, NJ, US) if not mentioned otherwise. Splenocytes of immunized or control mice (3×10^6^/well) were stimulated for 4 hours at 37°C and 5% CO_2_ with recombinant IN protein (10 µg/ml), an equimolar mixture of peptides representing mouse CD4^+^ and CD8^+^ epitopes Pool_MIN (Table 1) with a total peptide concentration of 10 µg/ml, luciferase peptide LUC (10 µg/ml), or medium alone. Concanavalin A (Con A, 2 µg/ml) served as a positive control. All stimuli were diluted in RPMI 1640 supplemented with 5% FBS, 100 U/ml penicillin, 100 µg/mL streptomycin, and 0.3 mg/ml glutamine (all from Gibco, Life Technologies Co), in the presence of GolgiPlug containing Brefeldin A.

Ten minutes before the end of incubation, anti-mouse CD16/CD32 antibody was added to block non-antigen-specific binding of immunoglobulins to Fcγ receptors. Surface staining was performed by incubating restimulated cells with Pacific Blue (PB)-conjugated anti-mouse CD8 and peridinin chlorophyll protein complex (PerCP)-conjugated anti-mouse CD4 antibodies. Cells were then fixed and permeabilized at room temperature for 20 minutes in 100 µl Cytofix/Cytoperm solution, washed with Perm/Wash buffer, and stained at 4°C for 30 minutes with fluorescein isothiocyanate (FITC)-conjugated anti-IFN-γ, allophycocyanin (APC)-conjugated anti-IL2, phycoerythrin (PE)-conjugated anti-IL4, and phycoerythrin-cyanine dye 7 (PeCy7)-conjugated anti-TNFα anti-mouse antibodies. Samples were acquired on a FACS Canto flow cytometer (BD Biosciences). The flow cytometry analysis was carried on FlowJo software (Tree Star Inc, Ashland OR, USA). The gating approach is illustrated in Fig. 6A. A general lymphocyte area was defined and single living cells within this population were defined by their expression of CD4 or CD8, and further, by production of IFN-γ, IL-2, IL-4, and TNF-α. Frequencies of CD8^+^ and CD4^+^ cells producing cytokines in response to IN- or Luc- specific stimulation were quantified, and values for unstimulated cells were subtracted.

### Software and Statistics

Integrase consensus was created using BioEdit software (Ibis Biosciences, Carlsbad, CA). The design of humanized genes included modeling of the RNA folding done using the web services at http://genomes.urv.es/OPTIMIZER, http://www.invitrogen.com, http://mfold.rna.albany.edu/, and http://genomes.urv.es/OPTIMIZER/. Radioactive signals were quantified using Image-QuantTM 4.1 (Amersham Biosciences Corp, Piscataway, NJ); Western blot images, using ImageJ (http://rsb.info.nih.gov/ij); and luminescent images, using Living Image software version 2.50.1 (Caliper Life Science). Continuous but not normally distributed variables, such as the antibody levels, number of cytokine-producing spot-forming cells, or radiance per area, were compared by the nonparametric Kruskal-Wallis and Mann-Whitney *U* tests. The Spearman rank-order correlation coefficient was calculated to characterize linear correlations between variables. Calculations were done using STATISTICA AXA 10.0 (StatSoft Inc., OK, USA).

## Supporting Information

Figure S1 **Expression of the IN genes in** ***E. coli*** **.** Expression of the consensus integrase of HIV-1 FSU-A strain (IN_a), inactivated consensus IN (IN_in), inactivated consensus IN with elvitegravir resistance mutations (IN_in_e3) cloned in the prokaryotic expression into pET15b vector in the *E. coli* strain BL21(DE3) carrying pRARE plasmid from the Rosetta (DE3) strain for minor tRNA synthesis. Data represent three independent expression experiments.
(TIF)

Figure S2 **IN-specific responses in PBMC of BALB/c mice immunized with IN genes analyzed by IFN-γ/IL-2 Fluorospot.** Dual IFN-γ/IL-2 Fluorospot was performed on PBMC of mice immunized with consensus IN (IN_a), inactivated consensus IN (IN_in), inactivated consensus IN with elvitegravir resistance mutations (IN_in_e3), or empty vector. PBMC were pooled group-wise, and stimulated with the Luc derived peptide (LUC), pooled peptides representing mouse CD4^+^ and CD8^+^ epitopes (Pool_MIN), and recombinant integrase of HXB2 (ProtIN) (see Methods for details). IN-specific *in vitro* secretion of IFN-γ (**A**), IL-2 (**B**) and dual secretion of IFN-γ/IL-2 (**C**). Responses represent the average number of signal-forming units (sfu) per mln cells expressed as the mean ± SEM for four mice in each group in two independent experiments.
(TIF)

Figure S3 **T cell responses elicited in BALB/c mice by Luc reporter gene visualized by multiparametric FACS.** *In vitro* stimulation by LUC peptide (Table 3) of the total CD4^+^ positive (**A, C**) and total CD8^+^ positive (**B, D**) cells resulting in the secretion of single (**A, B**) or multiple (**C, D**) cytokines given as % of responding cells. Data are expressed as the mean ± SEM for four mice in each group in two independent experiments. Statistical comparison by Kruskal-Wallis and F-tests of the percent of CD4^+^ (**E**) and CD8^+^ (**F**) T cells simultaneously secreting IFN-γ, IL-2 and TNF-α in mice immunized with IN genes or empty vector (each mixed with Luc reporter plasmid).
(TIF)

Figure S4 **Percent T-cells co-secreting IFN-γ/IL-2/TNF-α in response to IN-specific stimulation in the IN gene and vector-immunized mice.** Comparison by the Kruskal-Wallis and F-tests of the mean percent of CD4^+^ (**A**) and CD8^+^ (**B**) T cells simultaneously secreting IFN-γ, IL-2 and TNF-α after *in vitro* stimulation with MIN peptide pool (Table 3) in mouse groups immunized with plasmids encoding the consensus HIV-1 FSU-A integrase (IN_a), inactivated integrase (IN_in), inactivated integrase with elvitegravir resistance mutations (IN_in_e3) or empty vector. Analysis is performed in the data from two independent experiments.
(TIF)
