# Supplementary figures and images for: Consensus HIV-1 FSU-A Integrase Gene Variants Electroporated into Mice Induce Polyfunctional Antigen-Specific CD4+ and CD8+ T Cells
Source: PLoS One. 2013 May 8;8(5):e62720. doi: 10.1371/journal.pone.0062720 (PMC3648577; doi:10.1371/journal.pone.0062720)

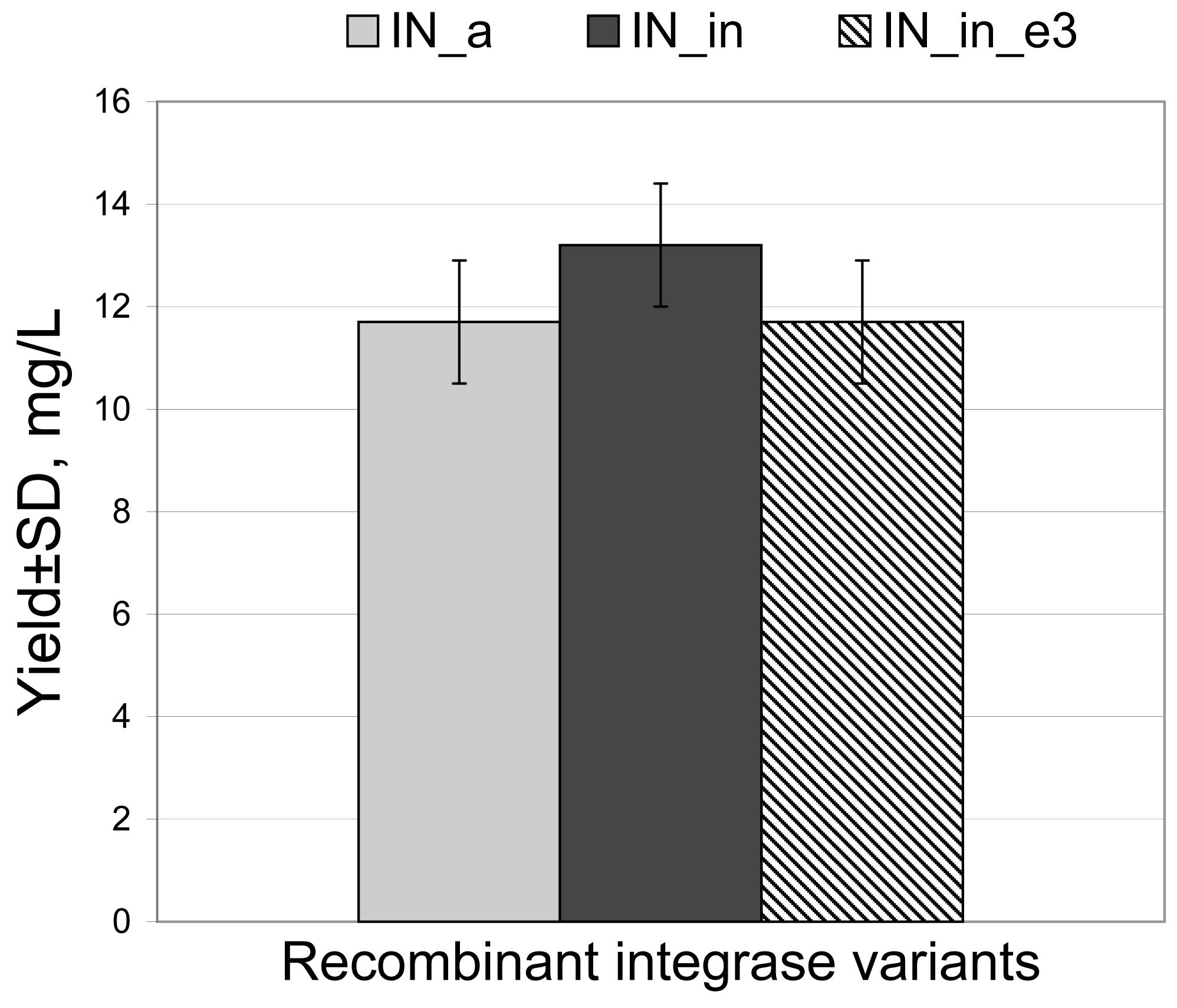

Supplement: Figure S1 — Expression of the IN genes in E. coli . Expression of the consensus integrase of HIV-1 FSU-A strain (IN_a), inactivated consensus IN (IN_in), inactivated consensus IN with elvitegravir resistance mutations (IN_in_e3) cloned in the prokaryotic expression into pET15b vector in the E. coli strain BL21(DE3) carrying pRARE plasmid from the Rosetta (DE3) strain for minor tRNA synthesis. Data represent three independent expression experiments. (TIF) [file pone.0062720.s001.tif]

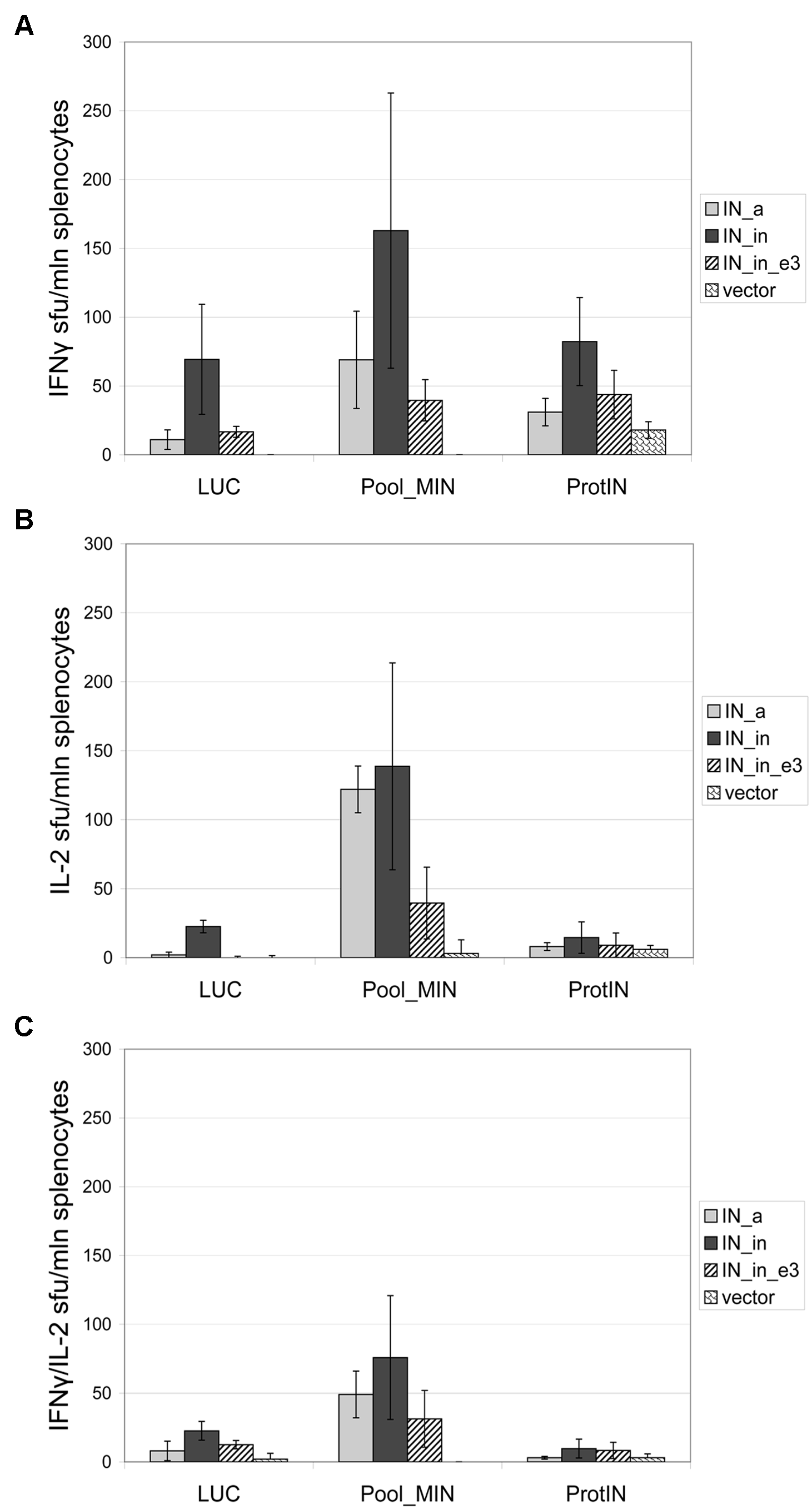

Supplement: Figure S2 — IN-specific responses in PBMC of BALB/c mice immunized with IN genes analyzed by IFN-γ/IL-2 Fluorospot. Dual IFN-γ/IL-2 Fluorospot was performed on PBMC of mice immunized with consensus IN (IN_a), inactivated consensus IN (IN_in), inactivated consensus IN with elvitegravir resistance mutations (IN_in_e3), or empty vector. PBMC were pooled group-wise, and stimulated with the Luc derived peptide (LUC), pooled peptides representing mouse CD4+ and CD8+ epitopes (Pool_MIN), and recombinant integrase of HXB2 (ProtIN) (see Methods for details). IN-specific in vitro secretion of IFN-γ (A), IL-2 (B) and dual secretion of IFN-γ/IL-2 (C). Responses represent the average number of signal-forming units (sfu) per mln cells expressed as the mean ± SEM for four mice in each group in two independent experiments. (TIF) [file pone.0062720.s002.tif]

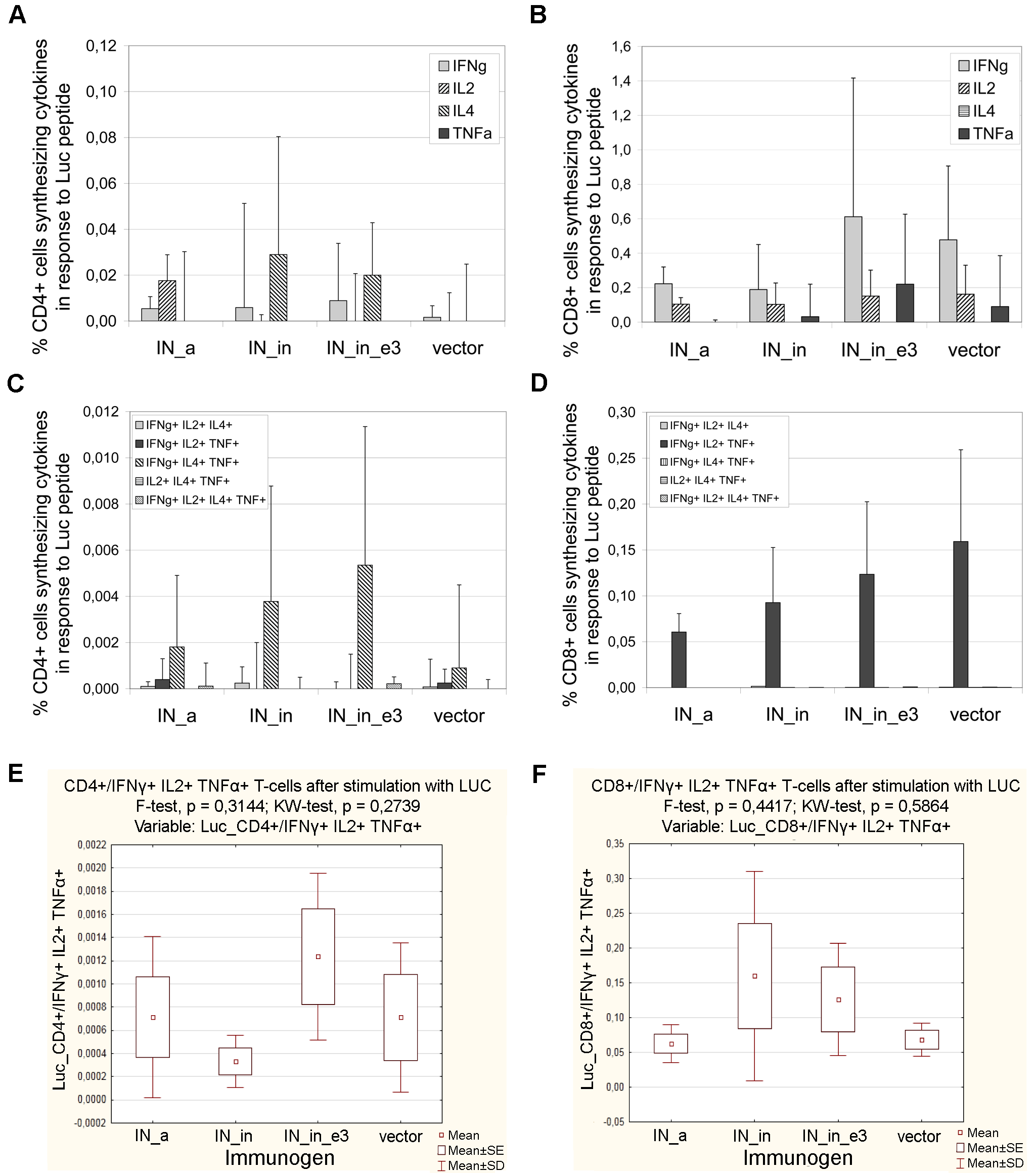

Supplement: Figure S3 — T cell responses elicited in BALB/c mice by Luc reporter gene visualized by multiparametric FACS. In vitro stimulation by LUC peptide (Table 3) of the total CD4+ positive (A, C) and total CD8+ positive (B, D) cells resulting in the secretion of single (A, B) or multiple (C, D) cytokines given as % of responding cells. Data are expressed as the mean ± SEM for four mice in each group in two independent experiments. Statistical comparison by Kruskal-Wallis and F-tests of the percent of CD4+ (E) and CD8+ (F) T cells simultaneously secreting IFN-γ, IL-2 and TNF-α in mice immunized with IN genes or empty vector (each mixed with Luc reporter plasmid). (TIF) [file pone.0062720.s003.tif]

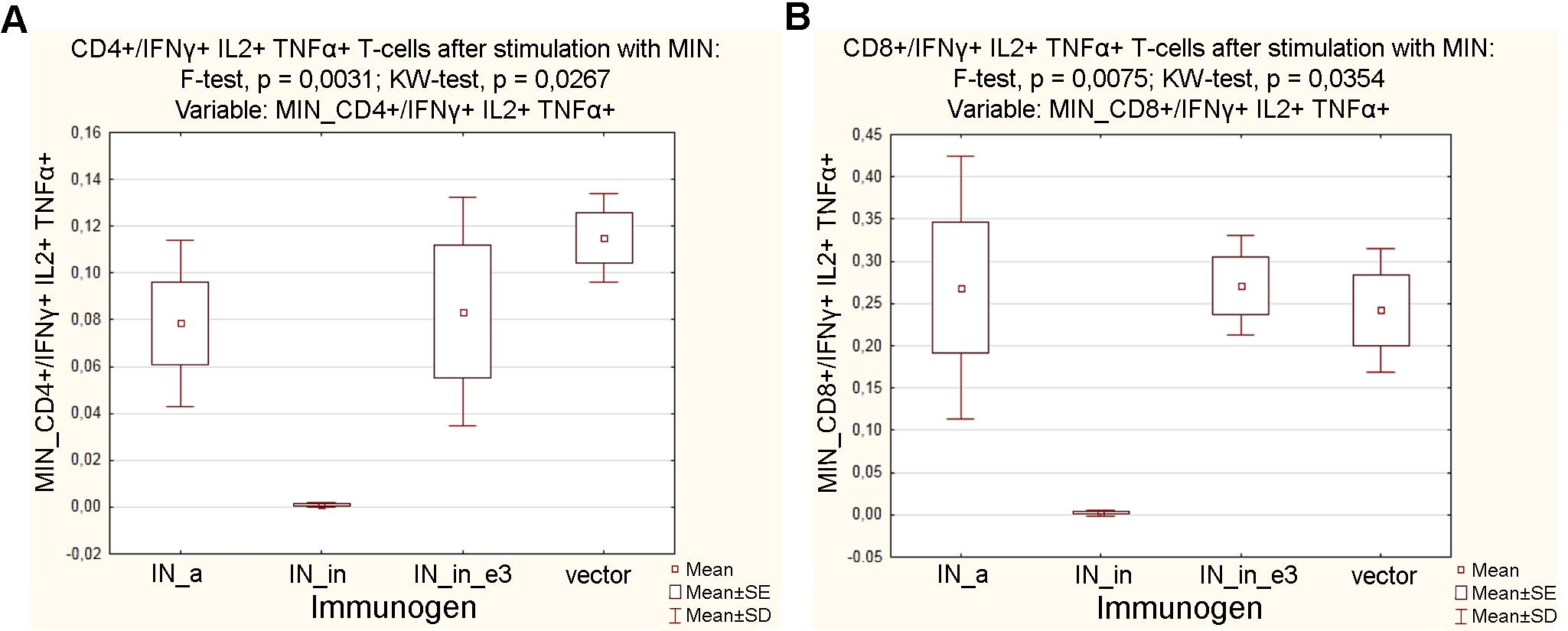

Supplement: Figure S4 — Percent T-cells co-secreting IFN-γ/IL-2/TNF-α in response to IN-specific stimulation in the IN gene and vector-immunized mice. Comparison by the Kruskal-Wallis and F-tests of the mean percent of CD4+ (A) and CD8+ (B) T cells simultaneously secreting IFN-γ, IL-2 and TNF-α after in vitro stimulation with MIN peptide pool (Table 3) in mouse groups immunized with plasmids encoding the consensus HIV-1 FSU-A integrase (IN_a), inactivated integrase (IN_in), inactivated integrase with elvitegravir resistance mutations (IN_in_e3) or empty vector. Analysis is performed in the data from two independent experiments. (TIF) [file pone.0062720.s004.tif]
